# Protein Import into the Endosymbiotic Organelles of Apicomplexan Parasites

**DOI:** 10.3390/genes9080412

**Published:** 2018-08-14

**Authors:** Natalia Mallo, Justin Fellows, Carla Johnson, Lilach Sheiner

**Affiliations:** 1 Wellcome Centre for Molecular Parasitology, University of Glasgow, 120 University Place Glasgow, Glasgow G12 8QQ, UK; natalia.mallo@glasgow.ac.uk (N.M.); 2128907J@student.gla.ac.uk (C.J.); 2 Genetics and Biochemistry Branch, National Institute for Diabetes and Digestive and Kidney Disease, National Institutes of Health, Bethesda, MD 20892, USA; justin.fellows@nih.gov

**Keywords:** apicoplast, mitochondrion, *Toxoplasma*, Apicomplexa, trafficking, import

## Abstract

The organelles of endosymbiotic origin, plastids, and mitochondria, evolved through the serial acquisition of endosymbionts by a host cell. These events were accompanied by gene transfer from the symbionts to the host, resulting in most of the organellar proteins being encoded in the cell nuclear genome and trafficked into the organelle via a series of translocation complexes. Much of what is known about organelle protein translocation mechanisms is based on studies performed in common model organisms; e.g., yeast and humans or *Arabidopsis*. However, studies performed in divergent organisms are gradually accumulating. These studies provide insights into universally conserved traits, while discovering traits that are specific to organisms or clades. Apicomplexan parasites feature two organelles of endosymbiotic origin: a secondary plastid named the apicoplast and a mitochondrion. In the context of the diseases caused by apicomplexan parasites, the essential roles and divergent features of both organelles make them prime targets for drug discovery. This potential and the amenability of the apicomplexan *Toxoplasma gondii* to genetic manipulation motivated research about the mechanisms controlling both organelles’ biogenesis. Here we provide an overview of what is known about apicomplexan organelle protein import. We focus on work done mainly in *T. gondii* and provide a comparison to model organisms.

## 1. Introduction

Endosymbiosis allows cells to acquire new functions by adding an endosymbiont with new biochemical “skills” and through evolving this symbiont into an organelle. For example, this is the route through which a proteobacterium became a mitochondrion [1]. This is also the route through which the ancestor of the eukaryotic clade named Chromalveolata acquired its secondary plastid [2]. In this latter secondary endosymbiotic event, one eukaryotic cell took in another eukaryote, a red algal cell, as its symbiont. The red algal symbiont evolved into a multi-membrane bound plastid. Apicomplexa is a phylum of parasitic protozoa that is included within the chromalveolates. The secondary plastid found in most known apicomplexans is named the apicoplast [3].

The evolution of an endosymbiont into an integral organelle is accompanied by gene-transfer from the endosymbiont genome to the host cell nucleus. This provides the host with control over symbiont function. Mechanisms that allow nuclear-encoded proteins to target to the symbiont must develop in parallel. Much of what we know about protein import mechanisms in plastids and mitochondria originates in studies performed in common model organisms. Yeast and humans are popular models of the eukaryotic opisthokont clade while green plants are the most commonly studied among the Archaeplastida clade. However, a growing number of studies exploring these basic biological questions in a variety of organisms representing divergent clades of the eukaryotic tree has been seen in recent years. This is partially thanks to the progression of technologies for culturing, genetic manipulation, and cell-biological analysis of more eukaryotic species. These studies enhance our appreciation of the true divergence of eukaryotic life, highlight universally conserved traits and separate them from organism- or clade-specific features.

Studies in apicomplexans contributed greatly to our understanding of cell biology in the chromalveolate clade. In the context of protein import into the endosymbiotic organelles, a theme is forming whereby the translocation machineries found in plant plastids and opisthokont mitochondria also operate in the apicoplast and in the apicomplexan mitochondrion. However, in most cases only the core components are identified, while any additional components are hypothesized to either be species-specific or missing altogether (e.g., [4,5,6]). Another emerging theme is that the chromalveolate secondary plastids re-tool conserved cellular mechanisms that are not known to have a role in plant plastid biogenesis for plastid compartment targeting (e.g., [7,8]). Below we review several examples of the conserved features and clade-specific features that define apicomplexan organelle protein import.

## 2. The Mitochondrion of Apicomplexan Parasites

Mitochondria and mitochondria-like organelles are nearly ubiquitous and are essential for most eukaryotes owing to their fundamental cellular functions. Historically, the major role described for mitochondria is the production of ATP through oxidative phosphorylation. However, it is now clear that mitochondria orchestrate other essential cellular roles such as calcium homeostasis, redox regulation and signaling and the biogenesis of cofactors such as iron-sulfur clusters. The latter is proposed to be conserved in all mitochondria and mitochondria-like organelles [9], and to be the mitochondrial “raison d’être” [10]. The essential role of mitochondria has been demonstrated for several apicomplexan parasites and mitochondrial functions are the target for several anti-parasitic drugs [11,12,13].

While it is generally a consensus that mitochondrial acquisition occurred once at the root of the tree of eukaryotes, mitochondria in different eukaryotic groups are highly divergent [14]. For example, the *Toxoplasma gondii* mitochondrion shows several divergent features compared to well-studied systems such as plants, fungi, and metazoans. *T. gondii* present a single large mitochondrion that only divides simultaneously with cytokinesis [15,16,17] while mammalian mitochondria may change their numbers independently of the cell cycle. The mitochondrion in intracellular *T. gondii* tachyzoites maintains a general morphology whereby the organelle is mostly found in close proximity to the cell periphery. This is in stark contrast to the mammalian mitochondria that can drastically change their morphology and cellular position. The *T. gondii* mitochondrial morphology is proposed to be mediated by membrane contact sites (MCS) to the parasite pellicles [17], and it was recently shown that this mitochondrion undergoes drastic morphological changes accompanied in reduced pellicle contacts when the tachyzoites are extracellular [17]. Another divergent feature is the markedly reduced mitochondrial genome size in apicomplexans and related organisms. Although the exact mitochondrial genome sequence of *T. gondii* is not known with certainty, the annotation from other apicomplexans and from the related Chromerida [18,19] suggests the presence of only three open reading frames, or less, in organisms of this group. The known mitochondrial genome sequences of apicomplexans encode apocytochrome b (*cob*), cytochrome c oxidase I (*cox1*) and III (*cox3*) genes [18]. This means that nearly the entire mitochondrial proteome is nuclear-encoded and imported from the cytosol. Likewise, other essential molecules such as transfer RNAs (tRNAs) need to be imported to support protein synthesis in the mitochondrion [20,21]. Surprisingly, evidence is also accumulating for differences in fundamental pathways previously considered ubiquitous. Good examples are provided by the recent description of the divergent features of major components of the mitochondrial electron transport chain and of the mitochondrial ATP synthase complex in *Toxoplasma* compared to the corresponding structures in the mitochondria of opisthokonts [22,23,24]. This first part of the review will focus on the differences and similarities between the mitochondrial protein import pathways of the well-studied opisthokonts and the apicomplexans *Toxoplasma* and *Plasmodium*.

### 2.1. Protein Import into the Apicomplexan Mitochondrion

#### 2.1.1. Signals Targeting Precursors to the Mitochondrial Sub-Compartments

**Figure 1:**
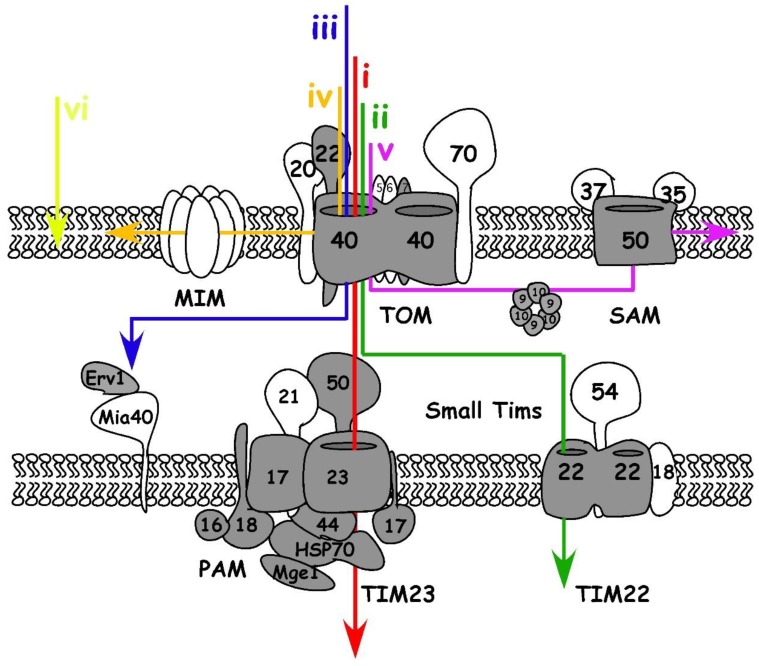
Complexes and route of protein entry into the mitochondrial compartments. The colored lines depict the routes of protein translocation into the different compartments of the yeast mitochondria (detailed in the “signals targeting precursors to the mitochondrial sub-compartments” section). Known components of the yeast translocation complexes are shown and those for which homologues are found in the genomes of *Toxoplasma* and *Plasmodium* spp. are in grey. MIM: mitochondrial import complex; PAM: pre-sequence translocase Associated Motor; SAM: sorting and assembly machinery complex; TOM: translocase of the outer mitochondrial membranes.

Mitochondria are bound by two membranes, the mitochondrial outer and inner membrane (OM and IM), that delimit two different compartments, the intermembrane space (IMS) and the matrix (Figure 1). The distribution of proteins into cellular compartments is dependent on protein translocation systems that decode transport signals embedded in the protein’s sequence or structure. Most mitochondrial proteins studied to date contain specific import signals that direct them down the suitable import pathway within the organelle. In yeast, six main signals are described (Figure 1) (see [25,26] for more detailed reviews): (i) an N-terminal pre-sequence directing proteins to the matrix, which is typically cleaved off there; (ii) precursors of non-cleavable multispanning IM proteins that contain internal targeting signals; (iii) proteins of the IMS with a cysteine-containing targeting signal; integral proteins of the OM are inserted through different pathways if they have (iv) alpha-helical transmembrane domains or if they are (v) β-barrel proteins. The last group (vi) consists of proteins with C-terminal membrane anchors (also named tail-anchored proteins).

The import signals governing apicomplexan mitochondrial protein trafficking were studied in detail in only a handful of cases. A canonical N-terminal cleavable pre-sequence (group (i) above) is recognized and targeted to the mitochondrion of *T. gondii* [27] suggesting conservation of this signal between apicomplexans and opisthokonts. However, while in yeast the signal is typically found within the first 15–55 amino acids [28,29], some *T. gondii* pre-sequences seem “recessed”. For instance, the amphipathic helix in the superoxide dismutase (TgSODB2) protein is found 25 amino acids away from its N-terminal pre-sequence [30] and this may also be the case for the *Toxoplasma* MutS homologue TgMSH [31]. Likewise, among a group of 27 proteins collected from the literature shown to be experimentally localized to the mitochondrion and predicted to reside within its matrix, 11 have predicted amphipathic helixes and cleavage sites that are found well downstream of the N-terminal 55 amino acids (Table S1). A recent study mapped a large proportion of the mitochondrial matrix proteome through the use of proximity tagging, identifying 461 putative matrix proteins [24]. Only 40% of these 461 proposed matrix proteins are strongly predicted to have a canonical N-terminal pre-sequence by the MitoProt II algorithm [24]. It remains to be experimentally determined whether this low frequency is due to divergence in targeting signals or due to false positives in the matrix proteome dataset [24]. We find that the data in Table S1 adds support to the former option. Further evidence supporting this possibility is provided by identifying that non-opisthokont organisms containing divergent mitochondrion-like organelles have matrix proteins with signals independent of the N-terminus [32,33].

*T. gondii* has mitochondrial proteins with the predicted presence of the other signal types. However, the signals governing those localizations have not been studied. Examples of the *T. gondii* mitochondrial proteins predicted to follow the type (v) signal include the β-barrel translocation pore TgSam50 [4], the protein import pore TgTom40 [6], and the putative Voltage Dependent Anion Channel (TGME49_263300) [34]. In agreement with this, MitoProt does not predict a canonical N-terminal pre-sequence in them (Table S1). Homologues of the group of chaperones named small Tims and of the sulfhydryl oxidase named Erv1 are identified [6,35] and predicted to be IMS residents (type (iii)). Mitochondria targeted tail-anchored proteins (type (vi)) are also found in *T. gondii*, such as the lysine acetyltransferase TgELp3 [36], where the localization was shown to be governed by the C-terminal transmembrane domain (TMD) and targeting sequence [37]. It is also important to note that dual targeting (e.g., to the mitochondrion and apicoplast or mitochondrion and cytosol) was also observed in apicomplexans [21,38], illustrating even more complexity to signaling in apicomplexan mitochondrion protein import.

#### 2.1.2. Proteins Gain Entry to the Mitochondrial Sub-Compartments via a Series of Translocases

A set of translocation complexes evolved to confirm the identification and delivery of proteins encoded in the nuclear genome to their suitable sub-compartment. The protein translocases operate in coordination to allow the proteins to reach different locations within the mitochondria. Below we discuss each translocation complex and compare what is known in the yeast model to what is known about apicomplexans.

#### 2.1.3. Protein Import in the Mitochondrial Outer Membrane 

The translocase of the outer mitochondrial membranes (TOM) is the first access gate for mitochondrial proteins. Yeast TOM complex contains one essential subunit, the channel Tom40, and 6 other subunits: the receptors Tom70, Tom20 and Tom22 and the small subunits Tom5, Tom6, and Tom7 (See [25] for a comprehensive review).

As in yeast [39,40,41,42], the *Toxoplasma* TgTom22 and TgTom7 are critical for the TOM complex assembly. Each is essential for parasite growth [6], and depletion of either of them results in the inability of mitochondrial matrix proteins to mature correctly [6]. In contrast, several divergent features of the *Plasmodium* [43] and *Toxoplasma* [6] TOM complexes are evident. Both parasite genomes lack identifiable homologues to the yeast Tom70 and Tom20 receptor proteins, and the N terminus of the apicomplexan Tom22 appears truncated. Only three TOM components are identified in *Toxoplasma*: TgTom40, TgTom22 and TgTom7, yet the *Toxoplasma* TOM complex is comparable in size to yeast TOM (≈400 kDa [6]) raising the possibility that components that are specific to the phylum replace the yeast homologues. Considering the above-mentioned potential divergence in the location of the pre-sequence targeting signals within the *Toxoplasma* mitochondrial matrix proteins, it is tempting to hypothesize co-evolution of this trait with parasite specific TOM receptors. Apicomplexans would not be alone, but rather join an emerging trend whereby representatives from each of the major eukaryotic lineages have different receptors [44,45,46]: Tom20 and Tom70 are unique to opisthokonts; plants have their unique Tom20 and mtToc64; and trypanosomatids (where the origin of the whole TOM-like complex is debatable [47,48,49]) have ATOM69 and ATOM46. In the context of this discussion, it is relevant to highlight that mitochondrial precursor proteins are imported into isolated mitochondria where the receptor component of TOM was modified via protease treatment [50]. Likewise, it is proposed that the last common ancestral TOM complex imported precursors without the aid of receptors [51]. We find that together these studies argue against a common receptor that is essential for transport through the translocon, and favor the role of receptors as lineage-specific facilitators of recognition and import.

After translocation through the TOM complex, there are different pathways protein precursors can take. The precursors of β-barrel proteins are transported by IMS small Tim chaperones to the Sorting and Assembly Machinery (SAM) complex that guides their integration into the OM [52]. In yeast, the core component of SAM is its pore, formed by the protein Sam50. Two other SAM complex subunits, Sam35 and Sam47, are described in yeast. Sam50 homologues are found in apicomplexan and related organisms. The mitochondrial localization of the *T. gondii* and *Plasmodium falciparum* Sam50s were validated experimentally [4]. However, Sam35 and Sam37 homologues are not identifiable in apicomplexans. SAM functional analysis in this phylum awaits further studies.

In yeast, precursors of OM proteins with alpha-helical structures seem to be inserted in a Tom40 independent way, and instead depend on the mitochondrial import complex (MIM) [53,54]. The yeast mitochondrial import protein Mim1 works in cooperation with Tom70. While homologues for neither can be found in the genomes of apicomplexans it is possible that a functional homologue exists, as is the case for the trypanosomatid *Trypanosoma brucei* [55].

#### 2.1.4. Import and Folding in the Intermembrane Space 

Precursors coming through the TOM complex may become IMS residents. There are three main types of IMS proteins (reviewed in [56]). The first will continue into the translocase of the IM, TIM23, after entry through TOM. However, instead of passing into the matrix, a “stop transfer” sequence will halt these proteins in TIM23. The proteins are either laterally inserted into the IM or undergo two proteolytic cleavage events: at the N-terminal pre-sequence and the stop transfer sequence. These cleavage events release the mature proteins into the IMS. The second type includes proteins that are permanently associated to binding sites at the IMS face of the IM or the OM. It is proposed that their import is energetically driven by those interactions with the membrane. The third type of IMS proteins are those with cysteines that can form disulfide bonds in the oxidizing IMS environment. These proteins are identified by the Mitochondrial intermembrane space Import and Assembly (MIA) machinery [57]. Two main components play a role in the MIA machinery. Mia40, is an oxidoreductase that identifies and forms disulfide bonds in these precursors resulting in their folding and retention in the IMS. The second component, Erv1, is a sulfhydryl oxidase which re-oxidizes Mia40, thus recycling it for another round of precursor folding [57]. Surprisingly, while apicomplexans and other chromalveolates possess homologues of known MIA substrates, a Mia40 homologue is not identifiable in their genomes and only Erv1 homologues have been identified [6,35]. Interestingly, absence of Mia40 but presence of Erv1 is also observed in the genomes of an unrelated group of protist parasites, kinetoplastids, which belongs to another non-opisthokont clade (excavates) [49,58]. This led to the suggestion that before the MIA pathway appeared in evolution; the IMS import pathway required only Erv1 for the function of the primordial route. It also suggests that evolutionarily earlier versions of Erv1 may have fulfilled the role of both MIA components or that a non-Mia40 homologue existed in those putative early versions that performed its function [59]. However, a first set of cross-species complementation studies between yeast and the excavate organism *Leishmania tarentolae* place doubt on this model [60]. It will be interesting to examine similar cross-species work between yeast and apicomplexans.

Since the mitochondrial inner membrane employs a set of transporters and carriers to allow the controlled passage of molecules, some of the precursors reaching the IMS will become integrated into the IM. In yeast, these hydrophobic carrier proteins are imported by IMS small Tim chaperones, such as Tim9 and Tim10, and are inserted into the inner membrane by Tim22 [61]. Comparative genomic studies suggest that apicomplexan genomes encode for the main insertase Tim22. Its expression and localization were recently confirmed [6], but none of the other components of this complex in yeast is identifiable in apicomplexan parasites.

#### 2.1.5. Protein Import through the Mitochondrial Inner Membrane and into the Matrix

TIM23 is the complex that mediates the import of proteins containing a matrix targeting pre-sequence into the matrix. In yeast, Tim23 is the pore of the complex and it is shown to function in a dynamic way whereby it associates with different proteins in the different stages of translocation. In yeast, Tim50, Tim17, Tim21, and Mgr2 are involved in the TIM23-TOM interaction at the early stage of translocation [62,63]. Tim44 and components of the motor complex, named the Pre-sequence translocase Associated Motor (PAM), play a role in the late stage of the ATP-dependent translocation of the precursor proteins into the matrix. *Toxoplasma* homologues of Tim23, Tim50, and the PAM subunit Pam18 were identified and demonstrated to localize to the parasite mitochondrion [6]. Furthermore, putative *Toxoplasma* homologues were identified for Tim17, Mgr2 and the full set of PAM components [6]. In addition to *T. gondii*, comparative genomics identified homologues of Tim23, Tim50, Tim17, and several PAM subunits in *Plasmodium* spp. [43,64]. These observations suggest conservation of the TIM23 machinery in apicomplexan.

#### 2.1.6. Role of Mitochondrial Import Components in Forming Membrane Contact Sites

Functional links between membranes of different cellular compartments, named membrane contact sites (MCS), have been observed in eukaryotic cells for decades. However, the molecules forming the tethers that facilitate MCS and the function of different MCS are only recently becoming known and have been mainly described in opisthokonts [65,66,67]. The association of TIM23 and TOM is an example of a tether complex that facilitates the MCS between the OM and IM. A major complex in yeast mitochondria is the mitochondrial contact site and cristae organizing system (MICOS) that plays dual roles: maintaining mitochondrial architecture and facilitating the exchange of molecules between the OM and IM. The interaction of yeast MICOS with TOM and MIA facilitates IMS protein import. Also, MICOS’ interaction with TOM and SAM is critical for stimulating β-barrel protein import. The central subunits of MICOS are conserved in Apicomplexa [68]. However, their function in this parasite has not been studied.

The TOM complex is also involved in forming MCS on the other side of the OM. For example, components of TOM are proposed to control the MCS between the ER and mitochondria [69,70]. While the study of MCS involving the apicomplexan mitochondrion is in its infancy [17], evidence for functional ER-mitochondrial contacts is emerging [34]. Whether TOM is involved in the control of these contacts in apicomplexans remains an open question.

## 3. The Plastid of Apicomplexan Parasites (the Apicoplast)

**Figure 2:**
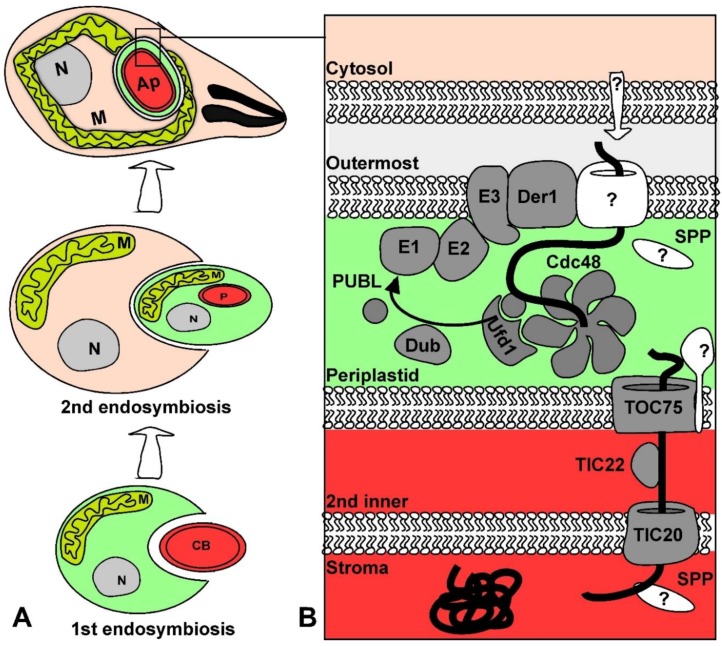
History of apicoplast acquisition, the resulting sub-compartments and the complexes involved in protein entry into the apicoplast compartments. The schemes are color coded to highlight the origin of each compartment (e.g., the PPC, corresponds to the algal cytosol (green)). (**A**) The scheme depicts the two endosymbiotic events that lead to the formation of the apicoplast. In the first event (bottom) a cyanobacterium (CB) is taken up by another cell. In the scheme, the host cell has a mitochondrion (M) and nucleus (N), however this is only one of a few models. In the second event, a eukaryotic cell with mitochondria (M) and a cell-nucleus (N) takes up a red algal cell along with its primary plastid (P) which evolved to a fully integrated secondary plastid in the apicomplexans—the apicoplast (AP); (**B**) Proteins participating in apicoplast protein import in each sub-compartment are depicted. Predicted components for which the apicomplexan homologues have not been identified, or unknown pathways are in white. A translocating protein from the outermost membrane to the stroma is depicted as a black bold line. In the PPC, the components of the endoplasmic reticulum associated degradation (ERAD) and ubiquitination pathways are shown: Der1; Cdc48; Ufd1; ubiquitin activating enzyme (E1); ubiquitin conjugating enzyme (E2); ubiquitin ligase (E3); deubiquitinase (Dub) and the plastid ubiquitin-like (PUBL) protein.

The apicoplast is a descendant of secondary endosymbiosis of a red algal symbiont with a heterotrophic eukaryote, which resulted in a four membrane-bound organelle [71] (Figure 2). While the original secondary endosymbiosis event that produced the complex plastids in the chromalveolate ancestor led to the acquisition of photosynthetic functions, the apicoplast has lost its photosynthetic properties. Nevertheless, the apicoplast is still necessary for parasite survival as it is the site of essential metabolic pathways including fatty acid, isoprenoid precursor, and heme biosynthesis [72]. The apicoplast is a prime therapeutic target for apicomplexan diseases due to its essential role and absence from mammals [73]. The amenability of *T. gondii* and *Plasmodium* spp. to genetic manipulation and the apicoplast’s potential as a therapeutic target provided the impetus for research that has generated insight into the mechanisms of secondary plastid biogenesis. 

Like the apicomplexan mitochondrion, which shows divergent features compared to canonical mitochondria, the apicoplast also has features that are divergent from other plastids. One example is the re-tooling of autophagy to support apicoplast biogenesis. Autophagy is a conserved process amongst eukaryotes that culminates in the formation of autophagosomes around proteins and organelles that will be digested by the lysosome to recycle cellular components. In opisthokonts this mechanism is controlled by the autophagy (ATG) related proteins ATG3, ATG7, and ATG12-ATG5. These proteins act in an enzymatic cascade to attach ATG8 onto phosphatidylethanolamine (PE) on autophagosomes, which is necessary for fusion with lysosomes (reviewed in [74]). However, in *T. gondii*, in addition to a putative role in autophagy under starvation conditions [75,76], ATG8 has taken up a divergent function. TgATG8 is localized to the cytoplasmic side of the apicoplast during standard growth conditions [77,78]. It was recently proposed that ATG8 physically links the apicoplast to the centrosome during division and that this is critical for proper apicoplast segregation [8,79]. Another divergent feature of the apicoplast is the re-tooling of an ER protein degradation pathway for protein import into the organelle, which we discuss below.

Like other plastids, the majority of apicoplast proteins are nuclear-encoded and must be imported across the four membranes of the apicoplast. A recent proteomics study estimated that 346 apicoplast proteins are targeted into the *P. falciparum* apicoplast stroma [80] emphasizing the expected robust import. This part of the review will focus on the signals and translocation complexes that regulate protein import across the four membranes that surround the apicoplast.

### 3.1. Protein Import into the Apicoplast

#### 3.1.1. Signals Targeting Precursors to the Apicoplast Sub-Compartments

Nuclear encoded plastid proteins carry signals for trafficking to the plastid. It has been proposed that positively charged targeting sequences were selected for by the electrochemical gradient generated by the electron transport chains of both mitochondria and plastids [81]. The membrane potential acts in synergy with the positive charge to aid in protein transport across the membrane via an electrophoretic effect [82,83]. In the case of primary plastids, like the plant chloroplast, proteins typically contain an N-terminal transit peptide that is necessary and sufficient for import into the plastids [84,85]. The primary sequence of transit peptides is not conserved and varies in length. However, transit peptides possess an overall positive charge and are enriched with the hydroxylated amino acids serine and threonine [86,87]. Nuclear encoded proteins of complex plastids, like the apicoplast, employ a positively charged transit peptide that is similar to transit peptides of primary plastids [88]. However, the net charge of complex plastid transit peptides is considerably higher than the net charge of transit peptides from organisms with primary plastids [81,89]. Protein import into the apicoplast is more elaborate than into primary plastids because there are four rather than two membranes that proteins must cross: the outermost, periplastid, second inner and innermost membranes (Figure 2). Therefore, most nuclear encoded apicoplast proteins have adopted a bipartite leader at their N-terminus, which consists of a signal peptide followed by a transit peptide. The signal peptide is thought to drive co-translational translocation into the ER and to be cleaved in the ER while exposing the transit peptide [90,91]. The transit peptide guides import into the apicoplast where it is subsequently cleaved by an unknown protease in the organelle [92].

Signals are also required to differentiate between proteins of the different compartments of complex plastids: the outermost, periplastid, second inner, and stromal compartments (Figure 2). Proteins destined to the stroma often have an aromatic amino acid (most commonly phenylalanine) at the +1 site of the transit peptide in the complex plastids of *P. falciparum* and the diatoms *Phaeodactylum tricornutum* and *Guillardia theta* [93,94,95]. The amino acid composition of transit peptides from 47 experimentally confirmed *T. gondii* apicoplast proteins were analyzed and displayed an enrichment of aromatic amino acids tyrosine and phenylalanine at the putative +1 sites of stromal proteins [4]. Mutagenesis studies of the stromal apicoplast protein acyl carrier protein (ACP) showed that a point mutation of phenylalanine at the +1 site to alanine resulted in ACP shifting localization to the periphery of the organelle [4]. This provided the first experimental evidence that a single amino acid is required for import into the inner compartments of *T. gondii*. However, this is not a consistent feature of stromal apicoplast proteins as many of the stromal proteins are missing this +1 aromatic amino acid. Moreover, deletions of the transit peptide of the stromal apicoplast protein ferredoxin NADP^+^ reductase did not change the localization of the protein [96]. This suggests possible alternative routes or mechanisms for compartmental protein sorting. It is also important to note that there are apicoplast proteins that lack the canonical bipartite leader altogether and instead have a recessed hydrophobic patch or transmembrane domains [97,98,99,100,101,102,103,104]. 

#### 3.1.2. Proteins Gain Entry to the Apicoplast Sub-Compartments via a Series of Translocases

Apicoplast proteins need to make it into the different sub-compartments (a catalogue of *Toxoplasma* apicoplast proteins with experimentally confirmed sub-compartment localization is found in [4]). The identification of the different translocation complexes allowing translocation to each compartment was aided by comparative analysis. The comparison to organisms with primary plastid, like land plants, helped identify the translocon of the second inner membrane. The comparison to other groups within the chromalveolates clade who share a common origin of their secondary plastid with apicomplexans, helped identify the translocon of the periplastid membrane.

#### 3.1.3. Transport to the Outer Membrane of the Apicoplast

The path through which nuclear encoded apicoplast proteins are transported from the ER lumen to and across the outermost membrane is still debatable. Originally, evidence in *T. gondii* and *P. falciparum* had suggested that trafficking from the ER to the apicoplast was independent of the Golgi apparatus. This was based on experiments whereby treatment with the trafficking inhibitor Brefeldin A and the addition of ER retrieval signals did not affect apicoplast protein transport [105,106]. Re-examination of the ER retrieval signals experiment within a study performed in *P. falciparum* found that the addition of ER retrieval signals to nuclear encoded apicoplast proteins resulted in reduced trafficking to the apicoplast and in reduced transit peptide processing [107]. This suggests that the Golgi apparatus may play a role in trafficking after all. A suggested model proposes that a putative transit peptide receptor in the Golgi captures proteins for further shipment to the apicoplast to reconcile the previous contradictory findings. However, this does not address the observed transit peptide processing after treatment with Brefeldin A, which suggests that import is still occurring after blocking Golgi dependent secretory processes. A simple resolution of these conflicting data is the existence of two different trafficking pathways. The discovery of non-canonical nuclear encoded apicoplast proteins that lack signal and transit peptides increases the probability of two trafficking mechanisms from the ER to the outermost membrane of the apicoplast [99,100,102,103,104,108]. These non-canonical apicoplast membrane proteins were also observed in large vesicles found near the apicoplast or in some cases merging with the organelle [100]. The argument for two trafficking mechanisms was strengthened by work illustrating that luminal apicoplast proteins are absent from these large vesicles and that the large vesicles with non-canonical apicoplast proteins seem to be independent of the Golgi [109]. Recently, it has been demonstrated that an apicoplast thioredoxin, TgATrx1, plays a role in ER to apicoplast outermost membrane trafficking. TgATrx1 is proposed to facilitate packaging of apicoplast proteins into vesicles forming at the ER [110]. The selectivity of TgATrx1 and the identity of the apicoplast proteins dependent on its function for their trafficking await further studies.

Phosphatidylinositol 3-monophosphate PI(3)P is a lipid that associates with endosomes and plays a role in trafficking these compartments to the lysosome in a majority of eukaryotes (reviewed in [111]). PI(3)P localizes to the apicoplast and to the large vesicles associated with non-canonical apicoplast proteins. In addition, overexpression of PI(3)P binding domains and the use of a PI3 kinase inhibitor resulted in apicoplast biogenesis defects and the accumulation of these vesicles around the apicoplast [112]. Therefore, it was proposed that PI(3)P was involved in vesicular trafficking of apicoplast proteins to the outermost membrane of the apicoplast [112]. However, conditional depletion of PI(3)PK and PIKfyve, two kinases that function in the synthesis of PI(3)P and PI(3,5)P_2_ respectively, suggest against a direct role of PI(3)P in trafficking. While an apicoplast morphological defect is observed under PI(3)PK or PIKfyve depletion, these mutants do not display apicoplast import defects as tested for both peripheral and luminal apicoplast proteins [113].

The mechanistic details of ER to apicoplast targeting are still elusive. The new insights on the alternative routes, on the involvement of phospholipids, and of redox regulators in the control of trafficking presents a broader picture than initially assumed. It also opens new questions with regards to the underlying mechanisms. The field is now equipped with a series of tools in the form of conditional mutants, well-established apicoplast activity assays and a broad group of experimentally identified markers for each compartment [4,80]. Future work relying on this knowledge will deepen our mechanistic understanding.

#### 3.1.4. The Periplastid Membrane and the Endoplasmic Reticulum Associated Degradation Complex

The mechanism of how proteins cross the second outermost membrane or the periplastid membrane (PPM) of complex plastids derived from a red algal endosymbiont was a surprising discovery. The PPM is thought to be derived from the plasma membrane of the endosymbiont taken up during the secondary endosymbiosis event [114], or from the ER of the ancestor host [115]. The secondary plastids of cryptomonads, which belong to the chromalveolate organisms, retained a relic of the ancestral endosymbiont nucleus. Sequencing this so-called nucleomorph revealed that it encodes homologues of the endoplasmic reticulum associated degradation (ERAD) pathway [116]. These ERAD components were shown to be duplicated in the nuclear genomes of several evolutionary related organisms containing complex plastids including *T. gondii* [97,117,118,119]. It was proposed that the ERAD proteins, normally involved in recognition of misfolded proteins in the secretory pathway and in shipment of misfolded proteins across the ER membrane into the cytoplasm for degradation, have been re-tooled for import across the PPM [116,120]. This hypothesis was experimentally validated when the *T. gondii* apicoplast homologue of Derlin1 (Der1_AP_), a central component of the ERAD pathway, was shown to be essential for protein import into the organelle [97]. The *T. gondii* apicoplast homologue of CDC48, the AAA ATPase that is crucial for pulling proteins across the membrane in the classical ERAD system, and the *T. gondii* homologue of CDC48’s cofactor, Ufd1, were also identified in the periplastid compartment (PPC) [97]. A CDC48_AP_ conditional mutant provided evidence that CDC48_AP_ is also essential for apicoplast biogenesis and import across the PPM [121].

One of the major components of the ERAD system is the ubiquitin pathway which polyubiquitinates misfolded proteins in the cytoplasm acting as a marker for degradation by the proteasome [120]. Ubiquitin is attached to substrates through an enzymatic cascade of proteins that consists of the ubiquitin activating enzyme (E1), ubiquitin conjugating enzyme (E2), and ubiquitin ligase (E3) [122]. A hypothesis was put forward whereby the ubiquitin machinery has been re-tooled to facilitate protein transport across the PPM [116]. This is supported by findings that ubiquitination of misfolded proteins is required for translocation across the ER membrane [123,124]. The *T. gondii* apicoplast homologues of the ubiquitin activating enzyme (E1_AP_) and the ubiquitin conjugating enzyme (E2_AP_) have both been identified. Conditional depletion of the *T. gondii* E2_AP_ component provided evidence that E2_AP_ plays a role in the control of protein import into the apicoplast [7]. Likewise, the plastid ubiquitin-like (PUBL) modifier, which the apicoplast ubiquitination machinery most likely utilizes, has been localized to the PPC. Interestingly, PUBL is not similar in sequence to ubiquitin or any other known ubiquitin-like proteins. Nevertheless, PUBL, like E2_AP_, is also essential both for parasite growth and for protein import across the PPM [121]. The question that remains is what is the mechanistic role of PUBL in the control of apicoplast protein import. Genetic complementation assays that characterized PUBL have provided some clues into its role. It has been shown that the C-terminal diglycine motif of PUBL is essential for its function. The C-terminal glycine motif is conserved amongst all ubiquitin-like proteins and is the motif that forms a covalent bond with lysine residues of substrates [125]. This suggests that PUBL may act as an ubiquitin-like protein and bind to substrates through its C-terminal diglycine motif. It was also demonstrated that canonical *T. gondii* ubiquitin sent to the PPC can complement PUBL mutants suggesting that PUBL acts in a similar fashion to ubiquitin [121]. Similarly, proteins destined to the complex plastid of the chromalveolate *P. tricornutum* need lysine residues at the leader sequence to cross the PPM, which provides indirect support to the ubiquitin based import model [126]. In addition, the *P. falciparum* protease PfOTU that belongs to the deubiquitinase family was recently determined to associate with the apicoplast and control protein import [127]. The absence of identified substrates for PUBL modification leaves the door open for alternative models for the role of PUBL in the apicoplast. 

#### 3.1.5. Crossing the Inner Two Membranes of the Apicoplast

The two innermost membranes of the apicoplast are evolutionary derived from the two membranes of the ancestral symbiont primary plastid. Primary plastids utilize the translocon of the inner and outer chloroplast membrane (TIC/TOC) protein complexes for transport across the innermost and outermost membranes respectively (recently reviewed in [128]). In agreement with this evolutionary history, *T. gondii* employs reduced TIC and TOC protein complexes for protein import across the apicoplast innermost membranes. Homologues of the plant Tic20 and Tic22 proteins belonging to the TIC translocon were localized to the apicoplast and were both shown to be essential for protein import into the organelle [5,129]. In primary plastids, Tic20 acts as an integral membrane protein of the pore complex and Tic22 functions as a chaperone [128]. It is suggested that the apicoplast homologues have synonymous functions [5,129]. A homologue of the main pore component of the plant TOC complex, Toc75, was also identified in chromalveolates despite its high sequence divergence from plant Toc75. The Toc75 homologue was initially identified in *P. tricornutum* via bioinformatic searches for an Omp85 family member. Electrophysiological studies of the *P. tricornutum* Toc75 homologue provided evidence for its role in protein translocation [130]. The *T. gondii* and *P. falciparum* Toc75 homologues were then identified and localized to the apicoplast [4]. It was further demonstrated that TgToc75 is essential for apicoplast biogenesis and protein import [4]. Additionally, apicoplast import assays revealed that the TgToc75 mutant resulted in the loss of import for stromal proteins, but does not have an import defect on proteins that reside in the periphery of the apicoplast. This evidence supports the model that Toc75 promotes translocation through the second innermost membrane.

Only a few homologues of the core TIC/TOC machinery out of the dozen units that participate in translocation in primary plastids (recently reviewed in [128]) have been identified in the apicoplast. This may indicate a reduced import machinery. Besides Toc75, the major members of the TOC machinery in primary plastids are the GTP binding receptors Toc34 and Toc159 that recognize the transit peptide of proteins [131]. It is proposed that in secondary plastids the TOC machinery does not require receptors to differentiate plastid proteins from cytoplasmic proteins, in the same way that primary plastid TOC uses receptors, because this step of recognition already takes place after the proteins have crossed the outermost and PPC membranes [132]. However, it is possible that receptors do exist and have not been identified yet. This may also be the case for Tic110, which has been identified in complex plastids of other organisms [132,133] but no homologues are identified in apicomplexans. Overall, experimental evidence has provided a model whereby *T. gondii* nuclear encoded apicoplast proteins are transported through the Toc75 and Tic20 translocons with the help of Tic22 to reach the stroma of the apicoplast.

## 4. Discussion: *Toxoplasma* Is a Strong Model for Organelle Import and Biogenesis

The mechanism of protein import is a fundamental aspect of mitochondrial and plastid biology. Comparative analysis between divergent organisms provides pivotal insight into the mechanistic biology governing protein import. While the road to understanding organelle protein import in *Toxoplasma* and other chromalveolates is still long, a theme of conservation of core components with opisthokonts emerges from the observations made to date. These studies provide support to phylogenetic based models that aspire to define the primitive translocases in the last common ancestor [51]. Such models contribute to our understanding of the evolution of organelles and provide a basis to mature hypotheses about the mechanistic roles of different translocation components.

Interestingly, each of the translocation components studied in *Toxoplasma* to date was shown to be essential for parasite growth in culture [4,5,6,97,129]. This is in contrast to observations from other organisms. For example, Tic22 is essential in *Toxoplasma* [129] while redundant in plant chloroplasts [134]. Likewise, Tom7 is essential in *Toxoplasma* [6] while non-essential in yeast mitochondria [41]. These findings point to reduced redundancy in the parasites’ protein import machinery compared with the homologous machinery from other organisms which highlights strength for studying these processes in *Toxoplasma*. This strength is further enhanced by the well-established molecular tools and microscopy analyses and by the recent organelle proteomics analyses [24,80]. Finally, *T. gondii* genes encoding proteins of the same pathway or structure tightly co-express [135]. Thus analysis of co-expression patterns is powerful for predicting new organelle proteins that were not identified by other methods [99]. The development of organelle isolation with sufficient purity and quantity to allow the establishment of in vitro functional studies and structural analyses is the next step in consolidating *Toxoplasma* as a strong model organism for organelle biology. Also, mitochondria organelle isolation may enable sequencing of the *Toxoplasma* mitochondrial genome, which is a crucial outstanding question in the field.

While we now know many of the components of the translocation complexes, the fine-tuning of translocases’ function and of the flow of proteins through the translocases remains unknown. Recent observations reveal that redox regulators control apicoplast protein folding and sorting, which affect apicoplast functions [110]. Whether and how this may serve as a link between apicoplast functions and cellular redox remains to be addressed. The interplay between cellular cues, organelle function and the role of protein import in translating cues to functional changes is an important aspect of endosymbiotic organelle biology that requires further understanding.

## Supplementary Materials

The following are available online at http://www.mdpi.com/2073-4425/9/8/412/s1, Table S1: Experimentally confirmed *Toxoplasma* mitochondrial proteins. The table consists of proteins for which there is experimental support for mitochondrial localization, either from microscopy of tagged proteins or from co-IP with known mitochondrial partners. * Mitochondrial sub-compartment is predicted according to the location of homologues in other studied organisms. ** Matrix location is predicted due to the method of identification (matrix proximity tagging).
