# Supplementary material for: Protein Import into the Endosymbiotic Organelles of Apicomplexan Parasites
Source: Genes (Basel). 2018 Aug 14;9(8):412. doi: 10.3390/genes9080412 (PMC6115763; doi:10.3390/genes9080412)
Supplement: Supplementary file 1 [file genes-09-00412-s001.pdf]

Table 1 - Experimentally confirmed *Toxoplasma* mitochondrial proteins. The table consists of proteins for which there is experimental support for mitochondrial localization, either from microscopy of tagging proteins or from co-IP with known mitochondrial partners. *Mitochondrial sub-compartment is predicted according to the location of homologs in other studied organisms. **matrix location is predicted due to the method of identification (matrix proximity tagging).

Name	Paper	Gene ID or accession	MitoProt - pobability of mito import	MitoProt cleavage site	Expected location*
HSP28	PMID: 16339717	AY650281.2	0.1143	NP	matrix
COX2A	PMID: 29043530	AAO27883	0.1817	19	IM
COX2B	PMID: 29043530	AAO27882	0.7209	26	IM
ClpB	PMID: 29156781	TGME49_268650	0.9284	64	matrix
TgPRELID	PMID: 28168222	TGGT1_254250	0.2139	NP	IMS
HSP60	PMID: 11163440	AF116462	0.9299	23	matrix
GAD	PMID: 23159057	TGME49_280700	0.791	35	matrix
SODB2	PMID:14576360	AY176062	0.2666	40	matrix
MutS	PMID:19291232	TGME49_218840	0.0044	NP	matrix
Sam50	PMID: 26381927	TGME49_205570	0.1246	NP	OM
MAFI	PMID: 24781109	TGGT1_220950	0.0285	NP	
TgMys	PMID: 28202940	TGME49_215430	0.5146	29	OM
RlmN	PMID: 27991712	TGME49_209790	0.0241	NP	TA
Elp3	PMID: 23878194	TGME49_305480	0.0054	NP	TA
FiS1	PMID: 27991712	TGME49_263323	0.0177	NP	TA
TgPyC	PMID: 28726641	TGGT1_284190	0.2579	NP	matrix
TgPEPCKmt	PMID: 28726641	TGGT1_289650	0.969	27	matrix
TgPSD1mt	PMID: 24429285	DQ450198	0.9847	97	matrix
TgFPPS	PMID: 17724033	AY196327	0.9761	150	matrix

ICAP18, ATP syntase d subunit	PMID: 22144892	TGME49_268830	0.9595	42	matrix
Hypothetical	PMID: 22144892	TGME49_249690	0.9984	110	
TgNDH2-I	PMID: 18786503	DQ211932	0.5819	24	matrix
TgNDH2-II	PMID: 18786503	DQ228957	0.9831	62	matrix
TgPyKII	PMID: 18326043	AB118155	0.1688	23	bimodal
TgPRX3	PMID:7784785	AY251021	0.6273	NP	matrix
TgSOD3	PMID:7784785	AY254045	0.9103	NP	matrix
TgTPX1/2	PMID:7784785	AY633702	0.98	168	bimodal
TgBCKDH	PMID: 25032958	TGME49_239490	0.4072	38	matrix
TgDHODH	PMID: 22580100	TGME49_210790	0.8899	39	IM
ICAP2 ATP synthase b subunit	PMID: 27594426 BioRxiv https://doi.org/10.1101/314385	TGME49_231410	0.9652	41	matrix
ICAP3	PMID: 27594426	TGME49_215430	0.6056	29	
ICAP6	PMID: 27594426	TGME49_260180	0.9094	63	
ICAP8	PMID: 27594426	TGME49_218940	0.0002	NP	
ICAP9	PMID: 27594426	TGME49_247410	0.2079	NP	
ICAP11	PMID: 27594426	TGME49_215610	0.8564	29	
ICAP14	PMID: 27594426	TGME49_255245	0.9252	21	
ICAP15	PMID: 27594426	TGME49_282180	0.9777	44	
Citrate-synthase I (CS1)	PMID:18336823	TGME49_268890	0.9991	87	matrix
Aconitase (ACN)	PMID:18336823	TGME49_226730	0.9936	119	matrix
Isocitrate-dehydrogenase I (IDH1	PMID:18336823	TGME49_313140	0.9129	166	matrix
Succinyl-CoA-synthetase alpha (SCSa)	PMID:18336823	TGME49_290600	0.7149	42	matrix

Succinyl-CoA-synthetase (ATP) (SCSb)	PMID:18336823	TGME49_309752	0.9559	57	matrix
Malate-dehydrogenase (MDH)	PMID:18336823	TGME49_318430	0.2217	72	matrix
FAD Malate-dehydrogenase (MDH-FAD)	PMID:18336823	TGME49_288500	0.9886	98	matrix
putative ATP-dependent hsl protease ATP-binding subunit hslU	BioRxiv https://doi.org/10.1101/320184	TGGT1_210730	0.9204	95	matrix**
RAP domain-containing protein	BioRxiv https://doi.org/10.1101/320184	TGGT1_213420	0.9499	131	matrix**
ATPase, AFG1 family protein	BioRxiv https://doi.org/10.1101/320184	TGGT1_217020	0.1463	13	matrix**
phosphoribosylpyrophosphate synthetase	BioRxiv https://doi.org/10.1101/320184	TGGT1_220100	0.5956	11	matrix**
hypothetical	BioRxiv https://doi.org/10.1101/320184	TGGT1_223500	0.1031	26	matrix**
hypothetical	BioRxiv https://doi.org/10.1101/320184	TGGT1_229620	0.9856	59	matrix**
aspartate aminotransferase	BioRxiv https://doi.org/10.1101/320184	TGGT1_248600	0.1823	NP	matrix**
TPR repeat region protein	BioRxiv https://doi.org/10.1101/320184	TGGT1_258100	0.9766	96	matrix**
protein kinase	BioRxiv https://doi.org/10.1101/320184	TGGT1_259710	0.9904	25	matrix**
hypothetical	BioRxiv https://doi.org/10.1101/320184	TGGT1_260840	0.7802	36	matrix**
hypothetical	BioRxiv https://doi.org/10.1101/320184	TGGT1_263080	0.8834	NP	matrix**
hypothetical	BioRxiv https://doi.org/10.1101/320184	TGGT1_263400	0.5318	48	matrix**
hypothetical	BioRxiv https://doi.org/10.1101/320184	TGGT1_263840	0.1633	NP	matrix**
hypothetical	BioRxiv https://doi.org/10.1101/320184	TGGT1_265360	0.9455	29	matrix**
hypothetical	BioRxiv https://doi.org/10.1101/320184	TGGT1_278250	0.0684	NP	matrix**

hypothetical	BioRxiv https://doi.org/10.1101/320184	TGGT1_278720	0.2222	NP	matrix**
hypothetical	BioRxiv https://doi.org/10.1101/320184	TGGT1_290460	0.998	54	matrix**
hypothetical	BioRxiv https://doi.org/10.1101/320184	TGGT1_300030	0.1404	NP	matrix**
hypothetical	BioRxiv https://doi.org/10.1101/320184	TGGT1_275650	0.5658	NP	matrix**
TgApiCox16	BioRxiv https://doi.org/10.1101/320184	TGGT1_265370			matrix**
TgApiCox30	BioRxiv https://doi.org/10.1101/320184<br>PMID: 27594426	TGGT1_297810	0.9486	85	IM/matrix**
TgCox2A	BioRxiv https://doi.org/10.1101/320184<br>PMID: 18036550	TGGT1_226590	0.1874	19	IM/matrix**
TgCox2b	BioRxiv https://doi.org/10.1101/320184<br>PMID: 18036550	TGGT1_310470	0.7121	26	IM/matrix**
TgCox5b	BioRxiv https://doi.org/10.1101/320184	TGGT1_209260	0.9872	75	IM/matrix**
TgApiCox18	BioRxiv https://doi.org/10.1101/320184	TGGT1_221510	0.9827	48	IM/matrix**
TgApiCox23	BioRxiv https://doi.org/10.1101/320184	TGGT1_262640	0.892	37	IM/matrix**
TgApiCox19	BioRxiv https://doi.org/10.1101/320184	TGGT1_247770	0.5905	27	IM/matrix**
TgApiCox24	BioRxiv https://doi.org/10.1101/320184	TGGT1_286530	0.9404	18	IM/matrix**
TgApiCox26	BioRxiv https://doi.org/10.1101/320184	TGGT1_306670	0.401	NP	IM/matrix**
Tom40	PMID:27458014	TGME49_218280	0.5333	NP	OM
Tom22	PMID:27458014	TGME49_255245	0.9252	21	OM
Tom7	PMID:27458014	TGME49_210255	0.9135	43	OM
Mpp-α	PMID:27458014	TGME49_202680	0.9985	64	matrix
Tim23	PMID:27458014	TGME49_214150	0.1705	NP	IM
Tim22	PMID:27458014	TGME49_225710	0.0468	NP	IM
Tim50	PMID:27458014	TGME49_283590	0.9955	127	IM
Pam18	PMID:27458014	TGME49_202810	0.9587	32	IM
hypothetical	PMID:25691595	TGME49_295370	0.8954	58	

ATP synthase OSCP	BioRxiv https://doi.org/10.1101/321620	TGME49_284540	0.986	34	matrix
ATP synthase F0 subunits alpha	BioRxiv https://doi.org/10.1101/321620	TGME49_310360	0.9422	16	IM
ASAP-11	BioRxiv https://doi.org/10.1101/321620	TGME49_290030	0.9902	37	
ASAP-14	BioRxiv https://doi.org/10.1101/321620	TGME49_254450	0.0579	NP	
ATP synthase beta subunit	BioRxiv https://doi.org/10.1101/321620	TGME49_261950	0.997	67	matrix

*according to location of homologs in other studied organisms.

**matrix location is predicted due to the method of identification.
